# Plant Growth‐Promoting Rhizobacteria and Bacterial Biocontrol Agents in Tomato Disease Management: Mechanisms, Applications, and Omics Perspectives

**DOI:** 10.1002/gch2.202500320

**Published:** 2025-11-17

**Authors:** Mateka Patience Modiba, Thomas Bell, Olubukola Oluranti Babalola

**Affiliations:** ^1^ Food Security and Safety Focus Area Faculty of Natural and Agricultural Sciences North‐West University Mmabatho South Africa; ^2^ Department of Life Sciences Imperial College London Silwood Park Campus Buckhurst Road, Ascot Berkshire SL5 7PY UK

**Keywords:** biological control agents, conventional farming, organic farming, PGPR, plant health, tomato diseases

## Abstract

Plant diseases, agricultural intensification, and climatic catastrophes such as drought have all has a significant impact on agricultural production in recent years. For decades, synthetic agrochemicals have been the primary tool for disease management and yield enhancement. However, their use poses significant environmental and health risks. There are many studies on plant growth‐promoting rhizobacteria (PGPR) and bacterial biocontrol agents (BCA) as eco‐friendly alternatives to synthetic agrochemicals. This review synthesizes current knowledge on the direct and indirect mechanisms by which PGPR and BCA enhance tomato growth and suppress pathogens. Although some of these PGPR and BCA are known, their mechanisms are not completely understood. Emerging omics approaches, which include genomics, transcriptomics, proteomics, and metabolomics, are highlighted as powerful tools for elucidating plant‐microbe interactions and guiding next‐generation biocontrol strategies. By critically examining overlapping mechanisms and applications, this review clarifies the complementary roles of PGPR, BCA, and “omics” and identifies research gaps for more consistent and scalable use in agriculture.

## Graphical abstract

Plant growth‐promoting rhizobacteria and bacterial biocontrol agents offer eco‐friendly alternatives to synthetic agrochemicals for tomato production. This review examines direct and indirect mechanisms that enhance growth and suppress pathogens, and highlights emerging omics tools that clarify plant–microbe interactions. By comparing mechanisms and applications, it identifies research gaps to guide next‐generation biocontrol strategies.

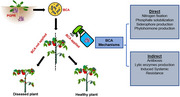

**Abbreviations**
PGPR: Plant growth‐promoting rhizobacteria
BCA: Biological control agents
spp: Species
Fe^3+^
: Iron
NH_3_
: Ammonia
IAA: indole‐3‐acetic acid
DAPG: 2,4‐diacetylphloroglucinol
ISR: Induced Systemic Resistance
ROS: Reactive Oxygen Species
DEGs: differentially expressed genes
RNA: Ribonucleic acid

## 1 Introduction

The world's anticipated population increase to 9.2 billion by 2050 would raise demand for agricultural products [1]. High food demand and a scarcity of new agricultural land development will require doubling crop yields, utilizing sustainable approaches in the future [2]. Additionally, plant pathogens pose a significant threat to agricultural production, as they cause diseases of economic importance. Therefore, there is pressure to use approaches that aid in plant health, development, and protection [3]. Farmers have extensively relied on synthetic fertilizers and chemical pesticides. However, these substances pose severe ecological threats, including soil and water contamination, the disruption of beneficial soil biota, and the promotion of pathogen resistance [4].

Soil health is crucial for crop development and establishment. Several studies have discovered that soil biota components, such as microbial community, abundance, variety, activity, and stability, are important indicators of soil quality [5, 6]. To protect the soil and plants, biological control has been suggested as the best option. A large range of microbial biological control agents (BCA) have been produced in recent decades for the management of plant diseases [7, 8]. The term biological control means using living organisms as “natural enemies” to eliminate or suppress plant diseases [9]. These natural enemies are usually microorganisms that use different approaches to reduce the population density of pathogens or enhance plant growth [10].

Some of these approaches include competing for space and organic nutrients; inducing the plant's defense mechanism; being an antagonist of plant pathogens; and producing antimicrobial metabolites [11, 12]. Some of the commonly known biological control agents are known to be plant growth‐promoting rhizobacteria (PGPR) [13]. There are many studies on plant growth‐promoting rhizobacteria (PGPR) having the potential to not only aid in plant growth but also in protection against phytopathogens and abiotic stress [14]. PGPR are present in many environments and form a huge part of the rhizosphere [15, 16, 17]. The genera *Bacillus* and *Pseudomonas* are the most extensively studied PGPRs due to their diverse mechanisms, though the complexity of their multi‐functional mode of action still requires in‐depth investigation [18, 19, 20].

Tomatoes are an economically important crop plant that could benefit from PGPR or BCA [6, 21]. Tomatoes (*Solanum lycopersicum* L.) are grown worldwide for their edible fruits and their health benefits [7, 22], In terms of production, South Africa has been ranked 39^th^ in the world. The consumption of tomatoes in South Africa is expected to rise to reach 543 thousand metric tons by 2026 (Food and Agriculture Organisation) [23]. Just like any other plant, tomatoes are prone to diseases, which are caused by multiple pathogens. The most common tomato diseases are bacterial wilt disease caused by *Ralstonia solanacearum* strains, and bacterial spot caused by *Xanthomonas spp* [24, 25]. The severity of these diseases increases under humid conditions, which results in devastating losses [26]. Due to global warming increasing the atmospheric humidity, the increasing world population, and disease threats, there is a dire need to find solutions to protect and sustain the tomato industry [27].

Understanding how management practices shape microbial communities is important for designing sustainable biocontrol strategies. This review focuses on how different farm practices alter the PGPR and BCA composition and synthesizes the direct and indirect mechanisms, applications in tomato protection, and future perspectives for their use in sustainable agriculture.

## 2 Different Types of Farming Practices

**FIGURE 1:**
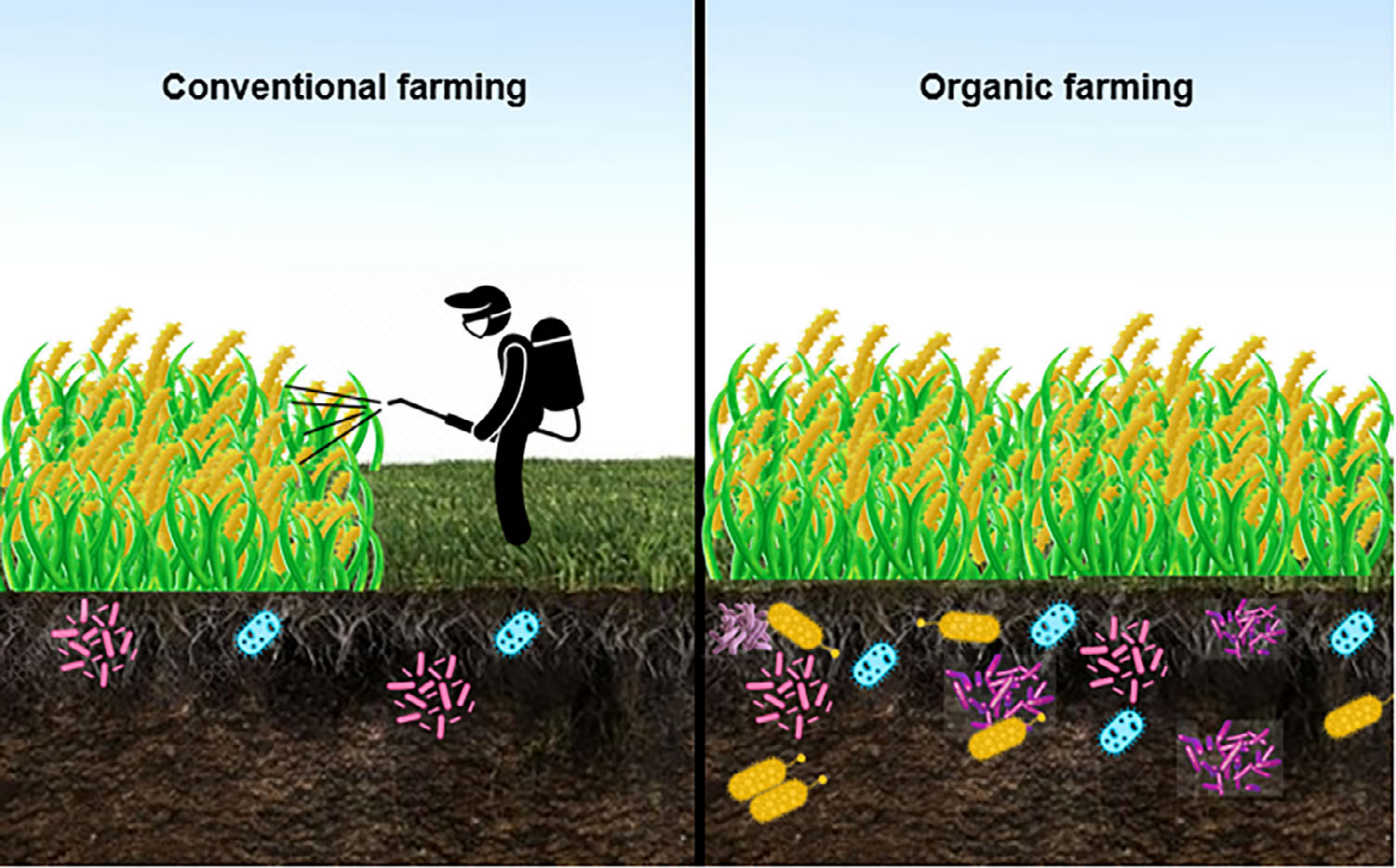
Comparison of microbial community diversity in soils under organic and conventional farming systems. Symbols indicate the relative abundance of dominant genera, including *Bacillus*, *Pseudomonas*, *Rhizobium*, and *Streptomyces*, among others.

In order to enhance production, promote plant health, and manage yields, conventional farming uses a lot of chemical fertilizers and pesticides [28]. But with the organic agricultural system, synthetic and chemical fertilizers are not used, nor are pesticides, which helps to lessen their damaging effects on the environment. Crop rotation, manual weeding, or mulching are utilized as alternatives to adding plant residue or livestock manure to improve soil fertility [29, 30]. More soil biodiversity and an abundance of macro‐ and microorganisms are also produced by organic farming (Figure 1) [31, 32]. Furthermore, crop diseases and pests pose a serious threat to agroecosystems, which can be more of an issue when growing organically because biotechnology and synthetic pesticides are not used [33]. Models and predictions indicate that by 2050, these dangers will become more prevalent, primarily as a result of the rapid climatic changes, which will particularly affect crops in hot, tropical regions. It is quite evident that when compared to other land management techniques, conventionally maintained croplands promote crop yields without requiring changes in land use or an expansion of farmland area [28]. However, in order to maintain a high yield, such agricultural practices require more chemical, fertilizer, and water inputs, which have an adverse effect on the ecosystems. As a result, the soil composition gets altered, usually in a negative way [34].

The most complex and diverse habitat on Earth is the soil, which is home to a vast variety of organisms [35]. Microorganisms are an essential component of soil ecosystems because they play a part in practically all soil functions and processes [36]. For instance, soil microbial communities have a key role in the cycling of nutrients and the breakdown of organic matter, as well as in the suppression of diseases and the defense of plants against environmental stresses [37, 38]. Soil biota, including the soil microbiome, must be conserved in the context of agricultural management in order to preserve agricultural productivity [39].

It is important to expand food production in order to meet the demand for food due to the anticipated rise in the global population [40]. Farmers must use intensive agricultural techniques that increase production in order to meet the demand on a worldwide scale [41]. The procedures that are typically used are chemical‐based and have a negative influence on the environment, animal health, and soil quality [42]. Farmers rely on mineral fertilizers and agrochemicals, which ultimately disrupt the soil's ability to cycle nutrients, harm beneficial insects, change the microbiome, pollute water systems by producing contaminated runoff, and contribute to climate change by causing variations in temperature and precipitation [43]. Additionally, the volatility in rainfall leads to an uneven distribution of rain, which causes soil erosion and degradation and has a negative impact on soil structure, productivity, and the environment as a whole [44]. Therefore, better sustainable agricultural practices are required.

Diverse microbiome groups are used in sustainable agriculture to protect plants through the use of biopesticides and biofungicides, and to acquire nutrients through rhizospheric, endophytic, or phyllosphere interaction [45]. When utilizing microorganisms, the environmental pollution is reduced, soil health and biodiversity are maintained, and natural resources are conserved against degradation of soil and water [39]. In order to meet the demand for food and fibre, sustainable agriculture integrates plant and animal products, such as farmyard manure and crop residue, while also improving both the environment's quality and the farmer's quality of life [46]. In order to decrease the use of off‐farm resources like agrochemicals, sustainability can be achieved by using animal manure and managing waste properly on the farm [47]. For instance, crop rotation with a variety of crops based on climatic conditions and source of water availability helps the farmers to minimize production risk and uncertainty, and improves soil ecological sustainability [48]. Practices used in sustainable agriculture include agroforestry, intercropping, crop rotation, green manuring, conservation tillage, cover crops, and the use of biofertilizers [48, 49, 50, 51].

By employing these techniques, the negative consequences are reduced. The most popular technique is the use of microbial‐based methods, including biostimulants, biopesticides, and biofertilizers, to manage plant diseases, increase crop output, and enhance soil health [52]. To enhance plant growth, health, and biomass, a particular group of microbes known as plant growth‐promoting rhizobacteria (PGPR) has been targeted [53]. Approximately one gram of the soil contains approximately four to six thousand distinct bacterial genomes [54]. They make the ideal partners for interacting with plants due to their varied metabolism and ability to utilize a large variety of chemicals as nutrition and energy sources [55, 56, 57, 58].

## 3 Direct and Indirect Mechanisms of Plant Growth Promotion and Disease Suppression

**FIGURE 2:**
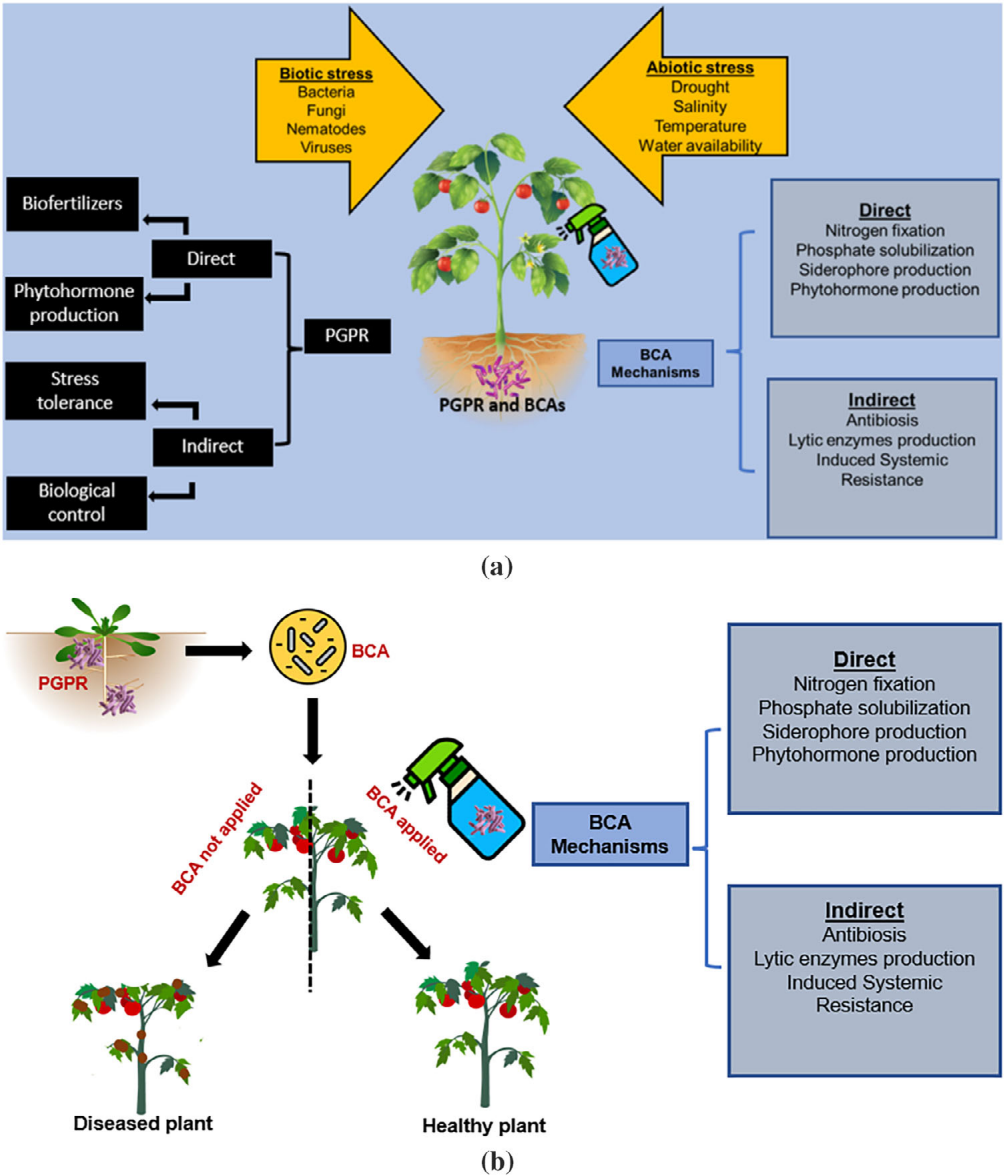
(A) Integrated overview of mechanisms used by PGPR and BCAs for plant growth‐promotion and disease suppression. (B) Direct and indirect effects of plant growth‐promoting rhizobacteria (PGPR) on plant development.

Plant growth‐promoting rhizobacteria (PGPR) are soil‐dwelling bacteria that colonize plant roots and stimulate plant growth [59, 60]. They boost plant health and growth by using their own metabolic products to directly influence plant metabolism to improve root development, increase enzyme activity and plant production, or suppress plant diseases [61, 62]. They also interact with the plant indirectly to protect it against diseases by competing with phytopathogens for limiting nutrients and space, controlling pathogens with aseptic‐activity chemicals, and generating systemic responses in host plants (Figure 2) [63]. They also assist plants in thriving and surviving in the face of abiotic stress by increasing plant fitness, stress tolerance, and pollution remediation [64, 65].

Research on the bacterial systems that promote and communicate with plants is ongoing [66]. There are numerous PGPR‐induced modifications in plants, and the stimulation of growth is probably the consequence of a complicated tangle of extra pathways that have an impact on both plant growth and nutrition [67]. Direct and indirect effects of PGPR on plant development are both favourable. PGPR influence on plant growth is a multigene process that is unique to each bacterium and plant involved [68]. Bacteria's direct techniques for promoting plant growth include increasing nutrient absorption and producing or controlling plant hormone levels [69]. Plant growth is influenced by PGPR's indirect processes, which also include a variety of systems for preventing or controlling plant diseases [70].

### 3.1 Contribution of PGPR and BCA to Plant Nutrition and Growth

Nitrogen, for example, is one of the most abundant and essential nutrients that contribute to plant growth and other metabolic functions [71]. It is, however, not in a form that plants can readily access. It must first be transformed to ammonia (NH_3_) before it can be utilized by plants. PGPR is involved in nitrogen fixation, the process by which nitrogen is converted to ammonia [72]. *Azotobacter*,  *Azospirillum*,  *Burkholderia*, *Bradyrhizobium*, *Enterobacter*, *Gluconacetobacter*, and *Stenotrophomonas* are some nitrogen‐fixing PGPR [73]. Phosphorus is the second most important element for plant growth. Phosphorus is essential in photosynthesis, plant metabolic processes, and energy transfer [74]. However, by mediating the mineralization of organic phosphorus in soil, these bacteria improve phosphorus availability in soil. Mineralization of organic phosphorus by microbes necessitates phosphorus solubilization, which is heavily influenced by soil pH [75]. Through a process known as rhizosphere acidification, these microorganisms alter soil pH by producing diverse organic and inorganic acids and other metabolites [76]. Phosphate‐solubilizing bacteria include *Rhizobium*, *Pseudomonas*, and *Bacillus* species [77].

Potassium, the seventh most prevalent element in the Earth's crust, is another essential macronutrient after nitrogen and phosphorus [78, 79]. It is essential for several activities in plants that are connected to metabolism, growth, and development. More than 80 enzymes involved in starch synthesis, nitrate reduction, photosynthesis, and other energy metabolic processes are activated by potassium [80]. A sufficient supply of potassium also controls an increase in plant vigour against numerous biotic stressors, diseases, and pests. In soil bacterial groups, *Bacillus mucilaginosus*, *B. edaphicus*, and *B. circulans* are the most efficient potassium solubilizers [81, 82].

Siderophores are tiny organic compounds produced by bacteria in iron‐deficient environments that enhance iron absorption. Iron is a micronutrient that is essential for all living things to survive [83]. The most common type of iron on Earth is Fe^3+^, but because it is weakly soluble, there is very little iron that is accessible to living things [84]. In iron‐limited soils, PGPR produce siderophores that increase iron availability to plants, enhancing growth while simultaneously limiting iron for pathogenic microbes and reducing their populations [53, 85, 86].

Production of phytohormones, which are organic substances that support plant growth and development, is another mechanism used by PGPR [87]. Gibberellins, cytokinin, abscisic acid, ethylene, auxin, and indole‐3‐acetic acid (IAA) are phytohormones that stimulate the overproduction of adjacent roots, which in turn increases nutrition and water intake and encourages the blooming of root cells [88, 89, 90]. Additionally, PGPR increases resistance to phytopathogens and generates certain metabolites that regulate phytopathogens in the root zone. A phytohormone known to facilitate plant‐microbe association is IAA [91]. Auxin production contributes to the stimulation of hormonal changes, the enhancement of root biomass, and the reduction of stomata size and density. Gibberellins and cytokinins help the plant grow its shoots and produce more root exudates [67, 92].

### 3.2 Contribution of PGPR and BCA to Plant Growth and Protection

Beyond plant growth promotion, PGPRs and BCAs directly suppress harmful microbes through the secretion of antibiotics, lytic enzymes, and volatile organic compounds [94]. Indirectly, these microbes can prime plant defense systems and outcompete pathogens for space and resources in the rhizosphere [95]. While these pathways overlap conceptually with PGPR mechanisms (Figure 2), the BCA context emphasizes pathogen suppression rather than general plant growth enhancement. This distinction is critical for understanding their application in targeted tomato disease management [96].

Antibiosis is one of the main biological control mechanisms, which is defined as the antagonism resulting from the toxicity of secondary metabolites produced by one microorganism for other microorganisms [97]. Some of those metabolites include lipopeptides, exoenzymes, and volatile organic molecules [98]. For instance, surfactin, which is a lipopeptide, is one of the antimicrobial substances that defend plants against phytopathogens by rupturing other species' cell membranes, integrating with the lipid layers, and lowering surface tensions [99, 100]. This inhibits the growth of metabolic activities of bacteria and other microorganisms.

These microbes use competition as one of its modes of action to attain better space and food availability. They end up competing for space and resources with other microbes in the rhizosphere due to the limited nutrient availability in the soil [101]. The competition for nutritional resources between pathogens and non‐pathogens is critical for controlling illness occurrence and severity. PGPR and BCA can swiftly colonize the soil and roots, exhausting the few available nutrients, leaving no room for pathogens to thrive [102]. They reduce the available nutrients at the wound site, preventing pathogens from germinating, growing, and infecting their hosts [10].

Plants can reach an enhanced defensive state of induced systemic resistance when stimulated correctly [103]. Some of the BCA produce secondary metabolites that induce resistance in plants against phytopathogens, by triggering a response that spreads systematically throughout the plant [104]. Many secondary metabolites involved in signal transduction, catalytic activities, and compounds such as salicylic acid, acetylsalicylic acid, and nitric oxide have properties that induce host plant immunity and enhance host resistance [105]. These compounds are responsible for the observed systemic acquired resistance after host plants are infected by pathogens and can be produced by many other non‐pathogenic microbes, such as rhizobacteria [106]. They are also commonly found in plant tissues but vary widely in extent among species, even genotypes within the same species. It is evident that some of these inducer compounds not only suppress plant diseases but also improve plant vigour, possibly due to the enhanced production of hormones [8, 107].

Some microbes are hyperparasites that produce antibiosis to directly kill pathogens or rely on pathogens for energy supply or living environments, while others may serve as competitors for niche and nutrients by releasing compounds or antimicrobials [108]. Some microbes are hyperparasites that produce antibiosis to directly kill pathogens or rely on pathogens for energy supply or living environments, while others may serve as competitors for niche and nutrients by releasing compounds or antimicrobials [8]. Some BCA colonize the plant root systems and improve the plant's fitness, growth, and development [109]. They employ mechanisms similar to those described for PGPR [15, 91, 110].

Establishing PGPRs and BCAs in the plant system before pathogen attack allows the host to maintain optimal fitness and effectively respond to potential stressors. The presence of these microbes in plants before pathogen introduction, therefore, mitigates negative impacts and enhances overall plant health (Figure 2) [111].

## 4 Applications of PGPR and BCA in Plant Protection

*Bacilli* are gram‐positive bacteria that have been extensively studied and are a highly promising choice for agricultural applications. They can interact both directly and indirectly with phytopathogens [112]. The primary biocontrol secondary metabolites generated by *Bacillus* are surfactin, bacillomycin D, sessilin, protease, chitinase, dimethyl disulfide, avermectin, prodigiosin, carbapenem group (1‐carbapen‐2‐em‐3‐carboxylic acid), serrawettins, althiomycin, oocydin A, 2,4‐diacetylphloroglucinol (DAPG), Phenazine, fengycin, and iturin A [113]. These metabolites are involved in antibiosis and the induction of Induced Systemic Resistance (ISR). Tomato and bean leaves, for example, that have high levels of surfactin and fengycin‐producing *B. subtilis* in their roots are more resistant to *Botrytis cinerea* illnesses than those that do not have *B. subtilis* [12]. Another study that looked at the interaction between *Bacillus* strains and strawberries found that BA strain S13‐3 induced ISR in strawberry leaves by generating iturin A and surfactins. They also activate the ISR by generating 2,3‐butadiene and 3‐hydroxy‐2‐butanone (acetoin) and boosting defense enzymes [114].

*Bacillus* also promotes plant health indirectly by delivering essential plant trace elements and nutrients to the plant. *B. subtilis* can convert air nitrogen into a form that plants can use [115]. They also boost plant growth and cell division by generating growth hormones or stimulating the plant to release its own growth hormones. *B. subtilis*, for example, causes cytokinin and ethylene homeostasis by generating acetoin (3‐hydroxy‐2‐butanone) and 2,3‐butanediol [116]. A study found that when *Arabidopsis thaliana* was inoculated with *B. subtilis*, the leaf size grew dramatically; however, the increase did not occur when the plant was inoculated with mutants of the cytokinin and ethylene pathways. As previously discussed in the PGPR section, *Bacillus* species produce IAA, which promotes plant growth by enhancing cell elongation, division, root development, and nutrient uptake [117]. This hormone also improves the quality and length of the main and lateral roots, as well as the intake of water and nutrients [118]. *Bacillus* species can modulate plant responses to abiotic stress, such as drought and salinity, with effects that may either enhance tolerance or, in certain cases, increase sensitivity depending on the plant species and bacterial strain [119].

Surfactin protects plants against diseases by breaking the cell membrane and integrating into the lipid layers, lowering surface tension [120]. In greenhouse testing, *Bacillus subtilis* 9407 was found to have high antibacterial action against *Acidovorax citrulli* and melon seedlings [121]. *Bacillus* produces proteases and chitinase, which aid in the breakdown of fungal cell walls [122]. *Bacillus* also produces volatile compounds that suppress spore germination and hyphal development of phytopathogens on agar plates in a contact‐independent way [123].

*Pseudomonas* is another type of bacteria that has been designated as a biological control agent. *Pseudomonas* is abundant and well‐known for its ability to manage soil‐borne illnesses, as well as being an excellent root colonizer [55]. Except for a few *P. syringae* strains, certain strains employed for biological control do not live well on above‐ground plant sections. *Pseudomonas* strains can promote plant growth by increasing mineral and nutrient availability and absorption via phosphate solubilization [125]. They also help to synthesize phytohormones and increase abiotic stress tolerance, both of which aid in root growth. *Pseudomonas* uses strategies such as antibiosis and competing for nutrients and space with other bacteria [126]. Some of the secondary metabolites produced by *Pseudomonas* are phenazine, pyoluteorin, rhizoxin, hydrogen cyanide, promysalin, i‐furanomycin, fit toxin, DAPG, and siderophore [12].

2,4‐diacetylphloroglucinol (DAPG) is a well‐conserved metabolite across *Pseudomonas* strains [127]. This polyketide antibiotic is primarily produced by *P. protegens* and *P. corrugata*, with a few strains from other taxonomic families. In *Arabidopsis*, DAPG induces ISR via promoting jasmonate‐ and ethylene‐mediated defense responses against the mildew pathogens *Hyaloperonospora parasitica*, *Pseudomonas syringae* pv. Tomato, and *Botrytis cinerea* [124, 128]. DAPG has also been shown to enhance root branching by inhibiting auxin‐dependent signalling, which stimulates amino acid exudation from plant roots [129].

*Serratia* are gram‐negative bacteria that include strains that are useful to plants as well as ones that are dangerous to people [131]. Prodigiosin, dimethyl disulfide, carbapenem group (1‐carbapen‐2‐em3‐carboxylic acid), serrawettins, althiomycin, and oocydin A are some of the secondary metabolites produced by this group. ISR in plants has been observed to be triggered by several strains, including *Serratia marcescens* CDP13. Wheat plants treated with *S. marcescens* CDP‐13 demonstrated a lower sensitivity to *Fusarium graminearum* disease in a water agar experiment. It has been claimed that some strains boost plant development by generating IAA. A greenhouse trial found that when plants were inoculated with *S. marcescens* NBRI1213, there was a significant increase in shoot length, shoot dry weight, root length, and root dry weight as compared to untreated control plants [132].

Prodigiosin is solely produced by four strains: *S. marcescens*, *S. plymuthica*, *S. nematodiphila*, and *S. rubidaea*. This metabolite serves as a chaotropic stressor, disrupting the bacterial plasma membrane and causing nutrients to be lost. They have the ability to change the hydrophobicity of the cell surface, which is necessary for these bacteria to cling to a variety of surfaces [12, 133]. Most *Serritia* strain studies are antifungal in nature. In a field study, *S. plymuthica* S13 and *Didymella bryoniae* were found to have an antagonistic effect on black rot in pumpkins. More study on bacterial plant diseases should be done [132].

*Streptomycetes* are gram‐positive bacteria found in soil that have attracted interest as biological control agents. Chitinase and avermectin are secondary metabolites generated by *Streptomycetes* strains [134]. They participate in competitive exclusion and the activation of host resistance mechanisms. They activate the plant's ISR by boosting the production of defense‐related components, localized cell death, and cell wall reinforcements, resulting in an intensified defense response [135]. When an oak tree was inoculated with *Streptomyces sp*. AcH505, pathogenesis‐related proteins were found to be upregulated. They can also activate the jasmonic acid‐ethylene and salicylic acid pathways, which can elicit ISR. For example, *S. lydicus* M01 treatment reduced the production of ROS and boosted the activities of antioxidases involved in ROS scavenging, indicating greater cucumber resistance to *Alternaria alternata* foliar disease [136]. *Streptomycetes* have been shown to enhance plant growth in ideal and severe climatic conditions, as well as protect plants from plant diseases. *Streptomycete avermectin* has been shown to kill cereal cyst nematodes. Some secondary metabolites are anti‐bacterial against *Ralstonia solanicearum*. These substances could be utilized to combat soil‐borne illnesses.

Purified chitinase from *Streptomyces lydicus* plays a function in the breakdown of chitin. WYEC108 has the ability to lyse the cell walls of a variety of phytopathogenic fungi, including *Pythium*, which can cause root rot in cereal crops. *Magnaporthe oryzae*, *Gaeumannomyces graminis* var. *tritici*, *Fusarium* species, and *Rhizoctani solani* can all be inhibited in vitro by *Streptomyces*. An endophytic strain VV/E1 of *Streptomyces* sp. and rhizosphere strains VV/R1 and VV/R4 of *Streptomyces* sp. were reported to have antifungal activity and decreased nursery fungal graft infections on grapevine plants [137]. Additionally, they create hydrolases, which act as mycoparasitics and prevent the spread of plant diseases. For instance, *Rhizoctonia solani* and *Macrophomina phaseolina* infections in *Phaseolus vulgaris* are reduced by *Streptomyces* CBQ‐EA‐2 and CBQ‐B‐8, which have chitinolytic, cellulolytic, and proteolytic activity [138]. Unfortunately, some *Streptomycetes* species create chemicals that hinder nitrogen‐fixing bacteria species in the roots from forming nodules. Other species, on the other hand, can encourage mycorrhizal development and nodulation while suppressing pathogen development. As a result, suitable biological control species must be carefully chosen and vetted [12].

*Pantoea* species are other bacteria that have been used as biological control agents. *Pantoea*‐containing biopesticides are registered and commercially accessible in Canada, New Zealand, and the United States [18]. Some of the mechanisms they use include competitive colonization, the creation of antimicrobial chemicals, and the activation of the plant's defense response. They produce secondary metabolites such as pantocins, herbicolins, microcins, and phenazines. *Pantaoea* species can also create N‐acyl‐homoserine lactone (AHL), which interferes with pathogen quorum sensing and improves plant environmental fitness, limiting pathogen development. Some strains, such as *P. agglomerans* EPS125 or CPA‐2, require direct cell‐to‐cell interaction to battle postharvest fungal infections without depending on antimicrobial chemical synthesis or nutritional competition [18, 139].

## 5 Future Perspectives and Emerging Strategies

The most recent method in the field of molecular microbiology is called “omics technologies,” and it entails the study of genomics, transcriptomics, proteomics, and metabolomics to determine the adaptability of plants to different stressful conditions [140, 141]. Understanding omics will make it easier to comprehend how an organism's phenotype is determined by the genetic information encoded in its codons [142]. Additionally, the use of omics approach in agricultural biotechnology has made it possible to identify several unique genes that play a role in crucial physiological traits of plants, like crop productivity and resilience to both biotic and abiotic stress [143, 144, 145, 146, 147]. Understanding the complex interactions between genes, proteins, and metabolites for the resulting phenotypes is made easier by omics techniques [148]. This integrated strategy relies heavily on a variety of chemical analytical techniques, in‐depth understanding of computer and bioinformatics analysis, and diverse biological disciplines, which result in crop protection and improvement [149, 150, 151].

Integrating genomics, transcriptomics, proteomics and metabolomics with advanced bioinformatics and predictive modelling can unravel complex plant‐microbe interactions. This suggests that coupling chemical‐analytical omics data with computational analysis and biological experimentation provides a powerful framework for improving crop protection strategies and accelerating the development of effective biocontrol agents [150, 152]. This has been demonstrated in some studies, where in one particular study it was demonstrated that the practical integration of proteomics and metabolomics with biological analysis revealed plant stress responses and yield‐related traits, illustrating how multi‐omics supports crop protection and improvement [153]. In another study, metabolomics, metagenomics, and computational analyses were able to uncover plant‐associated microbes, metabolite profiles, and crop phenotypes, and mechanisms crucial to crop improvement and biological control [154]. Collectively, these studies demonstrate that integrating chemical‐analytical omics, bioinformatics, and biological experimentation provides powerful insights for crop protection and improvement. Building on these integrative findings, the following research details specific experimental approaches and results that further validate and expand these strategies in practical biological control contexts.

For example, genes that are differentially expressed under specific conditions are revealed by transcriptomic analysis of plant‐associated bacteria utilizing RNA sequencing (RNA‐seq) technology or gene expression microarray techniques [155]. The majority of plant‐associated bacterial transcriptome research conducted to date has included isolating bacteria from their plant host. The majority of bacterial transcripts are housekeeping ribosomal RNAs, which presents a barrier for the study of bacterial transcriptomes in planta, because plant transcripts significantly outnumber bacterial transcripts [156]. Therefore, it is challenging to obtain bacterial mRNA transcripts at a concentration high enough for sequencing and differential expression studies [157].

As a result, it is difficult to get a sufficient concentration of bacterial mRNA transcripts for sequencing and differential expression investigation. Nobori and other researchers established two highly correlated techniques to enrich for the *Pseudomonas syringae* transcriptome in an *Arabidopsis* leaf infection model. During the first experiment, a novel separation buffer that stabilizes bacterial RNA was used. Before isolating RNA, bacterial cells were separated from plant cells using filtering and centrifugation. The second method used tailored probes to selectively deplete plant‐derived transcripts. It remains to be explored whether similar approaches can be applied to bacteria that live in the soil. RNA‐seq technique may also discover complex transcriptome control mechanisms such as gene operons, short noncoding RNA, antisense RNA, and riboswitches [156].

Many biological processes in plants depend on transcription factors (TFs). However, the roles of many TFs in tomatoes (*Solanum lycopersicum* L.) that control how the plant reacts to various stimuli are not well understood. A study using an RNA‐seq methodology discovered SlNAP1, a gene encoding NAC TF that was highly stimulated by several stressors. The evaluation of their response to biotic and abiotic stresses in tomato revealed that SlNAP1‐overexpressing plants exhibited significantly enhanced defense against two diseases: root‐borne bacterial wilt disease caused by *Ralstonia solanacearum* and leaf speck disease caused by *Pseudomonas syringae* pv. tomato (Pst) DC3000. Furthermore, tomato drought tolerance was significantly increased by overexpressing SlNAP1. Eventually, the fruit production of the SlNAP1‐overexpressing plants increased by 10.7%, despite their early vegetative stage being shorter than that of the wild‐type plants. Their findings show that SlNAP1 is a positive regulator of the tomato defensive response to numerous stresses, making it a promising breeding target for increasing crop output and stress tolerance [158].

*Cercospora arachidicola* causes early leaf spot disease, a deadly peanut disease that has significantly damaged peanut production and quality. A study was conducted to analyse the genome complexity of the novel strain *B. amyloliquefaciens* TA‐1 in order to uncover genetic factors that might be used as a biological control agent for the Early leaf spot illnesses. According to the findings, *B. amyloliquefaciens* TA‐1 suppressed the growth of the pathogen *C. arachidicola*, which causes early leaf spot. In‐vitro tests confirmed that the strain possesses broad‐spectrum antifungal activity, and a scanning electron microscope revealed that the *C. arachidicola* hyphae had been damaged and distorted. Seven gene clusters were found to express NRPS (non‐ribosomal peptide synthetase), which are enzymes that hinder cell wall synthesis and alter membrane function, among other things. Myxochelin, paenibactin, griseobactin, benarthin, and tailcyclized were among the siderophores coded. These compounds as previously mentioned, aid in the competition for iron nutrition, which is one of the most important biological control strategies of plant diseases [159].

*Botrytis cinerea* is a necrotrophic plant pathogen that affects over two hundred species. This pathogen is the cause of tomato Gray Mold. *Trichoderma harzianum* T4 is a well‐known biological control agent that is efficient against Gray Mold in tomatoes. A study was carried out to evaluate the mycoparasitism mechanism of *T. harzianum* T4. The expression of differentially expressed genes (DEGs) was examined at various time periods. For example, the results showed that hydrolase‐related genes were strongly expressed at 24 and 48 h, but not at 72 h. Cellulase‐related genes such as beta‐glucosidase and exo‐beta‐1,3‐glucanase, among others, were significantly elevated at 12, 24, and 48 h. With this information, it will aid in interpreting which biological control agent genes are crucial during which pathogen growth and development stage [160].

*B. amyloliquefaciens* MBI600 gene analysis was performed in a recent study. The investigation discovered 11 gene clusters that occupy 9% of its genome and are involved in antimicrobial metabolite production. Three gene clusters are involved in the nonribosomal synthesis of polyketides with antibacterial properties, such as difficidin, macrolactin, and bacillaene. Surfactin, bacillomycin, fengycin, and bacillibactin are all produced nonribosomally by another five clusters. Surfactins, as previously mentioned, are antibiotic chemicals that exhibit hemolytic, antibacterial, and antiviral actions by modifying membrane integrity, whereas fengycin is selective for filamentous fungi and inhibits phospholipase A2. The study showed that this strain has great potential to be a biological control agent [161].

The genome of *B. amyloliquefaciens subsp. plantarum* strain Fito_F321 isolated from grapevine leaves was studied. It has also been considered as the best model to research plant‐microbial interactions and their involvement in grapevine protection based on its genetic and physiological properties. The genome analysis revealed thirteen secondary metabolite gene clusters. It encoded four clusters of polyketide synthases, four clusters of nonribosomal peptide synthases, and one cluster of hybrid PKS‐NRPS. In addition, secondary metabolites such as ladderane, lantipeptide, and terpene cyclase were expected to be produced by the remaining four clusters. The findings of this investigation demonstrated that this strain can create important chemicals that aid in grapevine growth and protection [162]. Although the majority of these studies were performed on crops other than tomato, their integrative approaches provide valuable insights that are readily transferable to tomato disease management.

## 6 Conclusions

Agriculture is a vital component of the global economy. A variety of pathogenic microorganisms and abiotic stress have an impact on a wide range of agricultural crops. Due to abiotic/biotic stress, farmers rely on chemical pesticides to control diseases and synthetic fertilizers for growth. However, biological control agents are regarded as the ideal alternative technique for controlling pathogenic microorganisms, enhancing growth, and stress tolerance. Biological control agents have been studied over the years, but further research is needed. This review highlights the necessity of developing biological control agents with multi‐stress tolerance and efficacy, moving beyond single‐stress applications (e.g., disease) to address complex challenges like drought and salinity. Furthermore, it demonstrated how conventional farming has a less diverse microbial community. Because most farms in South Africa use the conventional approach, there is an urgent need to add biological control agents to promote healthy plant development and protection. While the complete elimination of chemical approaches in modern agriculture is impractical, a significant and conceivable reduction in their reliance is attainable through the adoption of biological agents.

## Conflict of Interest

The authors declare no conflict of interest.
